# The Peroxisomal *3-keto-acyl-CoA thiolase B* Gene Expression Is under the Dual Control of PPAR*α* and HNF4*α* in the Liver

**DOI:** 10.1155/2010/352957

**Published:** 2011-03-06

**Authors:** J. Chamouton, F. Hansmannel, J. A. Bonzo, M. C. Clémencet, G. Chevillard, M. Battle, P. Martin, T. Pineau, S. Duncan, F. J. Gonzalez, N. Latruffe, S. Mandard, V. Nicolas-Francès

**Affiliations:** ^1^Centre de Recherche, INSERM U866, LBMN 6, Boulevard Gabriel, 21000 Dijon, France; ^2^Laboratoire de Biochimie Métabolique et Nutritionnelle (LBMN), Faculté des Sciences Gabriel, Université de Bourgogne, 21000 Dijon, France; ^3^INSERM U744, Laboratoire d'Épidémiologie et Santé Publique, Institut Pasteur de Lille, 1 Rue du Professeur Calmette, BP 245, 59019 Lille Cedex, France; ^4^Laboratory of Metabolism, Division of Basic Sciences, National Cancer Institute, Bethesda, MD 20892, USA; ^5^Lady Davis Institute for Medical Research, McGill University, 3755 Côte Ste. Catherine Road, Montreal, QC, Canada H3T 1E2; ^6^Department of Cell Biology, Neurobiology and Anatomy, Medical College of Wisconsin, Milwaukee, WI 53226-0509, USA; ^7^Laboratoire de Pharmacologie et Toxicologie, UR66, INRA, 31931, Toulouse, France

## Abstract

PPAR*α* and HNF4*α* are nuclear receptors that control gene transcription by direct binding to specific nucleotide sequences. Using transgenic mice deficient for either PPAR*α* or HNF4*α*, we show that the expression of the peroxisomal * 3-keto-acyl-CoA thiolase B* (*Thb*) is under the dependence of these two transcription factors. Transactivation and gel shift experiments identified a novel PPAR response element within intron 3 of the *Thb* gene, by which PPAR*α* but not HNF4*α* transactivates. Intriguingly, we found that HNF4*α* enhanced PPAR*α*/RXR*α* transactivation from TB PPRE3 in a DNA-binding independent manner. Coimmunoprecipitation assays supported the hypothesis that HNF4*α* was physically interacting with RXR*α*. RT-PCR performed with RNA from liver-specific HNF4*α*-null mice confirmed the involvement of HNF4*α* in the PPAR*α*-regulated induction of *Thb* by Wy14,643. Overall, we conclude that HNF4*α* enhances the PPAR*α*-mediated activation of *Thb* gene expression in part through interaction with the obligate PPAR*α* partner, RXR*α*.

## 1. Introduction

Peroxisomes are essential organelles for various metabolic pathways including *β*-oxidation of very long-chain fatty acids (VLCFAs), prostaglandins, and leukotrienes as well as detoxification of xenobiotics and reactive oxygen species. Besides catabolic events, biosynthesis of cholesterol, bile acids, dolichol, and ether lipids (plasmalogens) also occurs within the peroxisomal matrix. While both mitochondria and peroxisomes are the main sites for cellular fatty acid degradation by oxidation, *β*-oxidations by these two organelles differ substantially in the substrate specificity. Whereas mitochondria mainly oxidize short, medium, and most long-chain fatty acids, peroxisomes preferentially oxidize very long straight-chain fatty acids (VLCFAs) (C > 20) and branched-chain fatty acids (BCFAs) [1].

Peroxisomal *β*-oxidation of VLCFAs can be divided in four main steps: dehydrogenation, hydration, oxidation, and thiolytic cleavage. While different enzymes are involved in the different biochemical reactions, thiolytic cleavage is accomplished by three enzymes in rodents, namely, 3-ketoacyl-CoA thiolase A (ThA), 3-ketoacyl-CoA thiolase B (ThB, EC:2.3.1.16), and Sterol Carrier Protein X/Sterol Carrier Protein 2 thiolase (SCPx/SCP2). 

In rodents, the *Tha *and *Thb* genes encode two distinct proteins that differ by 9 amino acids and display similar substrate specificity *in vitro *[2]. However, the *Tha* and *Thb* genes do not exhibit overlapping expression patterns [3]. While *Tha* is ubiquitously expressed, *Thb *is mainly present in liver and kidney [4]. The difference in tissue distribution suggests that ThA and ThB may have different biological activities. While ThB is assumed to be important for VLCFA metabolism, the exact function of this protein *in vivo* remains to be established. Although *Thb* ^−/−^ animals are phenotypically indistinguishable from wild-type littermates under normal conditions, feeding the potent peroxisome proliferator activated receptor alpha (PPAR*α*, NR1C1) agonist Wy14,643 (Wy) led to enrichment of the *n*-7 and *n*-9 medium chain unsaturated fatty acids (MUFAs) in the liver [5]. 

PPARs are ligand-activated nuclear hormone receptors involved in the regulation of numerous processes, including glucose, amino acid and lipid metabolism, inflammation and wound healing [6–8]. The three PPAR isotypes (*α*, *β*/*δ*, and *γ*) each regulate a distinct set of target genes by binding to DNA sequences consisting of two repeats of the consensus sequence AGGTCA separated by one nucleotide (Direct Repeat 1, DR1). DR1 sites specifically bound by PPARs and their dimerization partner retinoid x receptor (RXR) are also known as peroxisome proliferator response elements (PPREs) and are present either in the promoter region and/or intronic sequences of genes [9, 10]. The role of the nuclear receptor PPAR*α* in hepatic fatty acid oxidation has been well documented [11]. PPAR*α* serves as a nuclear receptor for fatty acids and is activated by the fibrate class of drugs, which are used in the treatment of dyslipidemia (low plasma HDL/high triglycerides). Transcriptional control of *Tha* and *Thb* by PPARs in the liver has been shown previously and involves a functional PPRE (TB PPRE2) in intron 3 of the *Thb* gene [12].

In addition to PPAR*α*, the liver-enriched transcription factor hepatocyte nuclear factor-4*α* (HNF4*α*, NR2A1) also plays a pivotal role in glucose, amino acid, and lipid metabolism. Because HNF4*α* is also known to recognize DR1-binding sites, PPAR*α* and HNF4*α* share common target genes, as previously shown for *glycogen synthase-2*,* acyl-Coa thioesterase I*, and *ornithine transcarbamylase* [7, 13, 14]. Another putative candidate for dual PPAR*α* and HNF4*α* regulation is* Thb.* A DR1 sequence (TB PPRE1) found in the promoter of *Thb* was shown to be bound by both PPAR*α* and HNF4*αin vitro* [15]. To confirm the *in vivo* relevance of this finding, the present study assesses the regulation of *Thb *by the nuclear receptors PPAR*α* and HNF4*α in vivo*, using* PPARα* ^−/−^ and hepatic HNF4*α*-null mice, respectively [16–18]. These *in vivo *models in combination with cell culture tools provide evidence for the involvement of HNF4*α* in Wy induction of *Thb* by interacting with the PPAR*α* dimerization partner retinoid x receptor alpha (RXR*α*) at a nonconventional PPRE located within intron 3 of the *Thb* gene.

## 2. Materials and Methods

### 2.1. Animal Experiments

Nine-week-old C57BL/6J PPAR*α*-null male mice and age-matched WT mice were used. Mice were kept in cages at 22°C, with equal periods of darkness and light and had free access to water and food containing 4.3% (w/v) lipids (U.A.R.A-03, Epinay sur Orge, France). For the pharmacological intervention, wild-type (WT) and PPAR*α*-null mice were fed by gavage for 8 days with Wy (30 mg kg^−1^·day^−1^ from Sigma). Control animals received the vehicle alone (3% arabic gum). Six-week-old liver-specifically HNF4*α*-disrupted (HNF4*α* ∆L) and control (HNF4*α* F/F) mice were fed a grain diet with or without (0.1% w/w) Wy for 5 days. Animals were sacrificed by cervical dislocation and tissues were rapidly snap-frozen in liquid nitrogen before storing at −80°C. Ethical considerations: *in vivo* studies were conducted under EU guidelines for the use and care of laboratory animals and were approved by an independent ethics committee. 

### 2.2. Cell Culture

COS-7 cells were grown as previously described [12, 15]. Briefly, COS-7 cells, mouse hepatoma Hepa 1.6 and human HeLa cells were routinely grown in Dulbecco's Modified Eagles Medium (DMEM) medium supplemented with 10% FCS and fetal calf serum (FCS) were from PAN Biotech GmbH. Rat hepatoma H4IIEC3 cells were grown in DMEM/HAM'S F-12 (1/1) medium supplemented with 5% FCS. All cells were grown in absence of antibiotics in the culture medium. Regular testing for mycoplasma contamination was performed with a PCR-based test.

### 2.3. Isolation of Total RNA and Northern Blotting Experiments

Total RNA was extracted from 50 mg from liver using TRizol reagent according to the method specified by the supplier (InVitrogen). Total RNA (15 *μ*g) was resolved on 1% agarose gels containing 6% (v/v) formaldehyde and transferred to Hybond-N membranes (Amersham Biosciences). Filters were hybridized overnight with ^32^P-labeled cDNA probes. Thb and 36B4 probes were previously described [3]. The Acox-I DNA probe was obtained by RT-PCR from mouse liver total RNA and verified by sequencing.

### 2.4. Isolation of Total RNA, Reverse-Transcription and Conventional PCR

*Hnf4* ^loxP/loxP^ *Alfp.cre *mutant mouse embryos [19] and *Hnf4* ^loxP/loxP^ *Alfp.cre* adult mice [17] were previously reported elsewhere. Total RNA from 18.5-d.p.c and adult livers were extracted using Qiagen's RNeasy mini kit following the manufacturer's protocol. Contaminating genomic DNA was removed using 10 u RNase-free-DNAse I/*μ*g RNA. cDNA was synthesized using MMLV-RT (Invitrogen) with dNTP (0.5 mM) and random hexamer primers (5 *μ*M). These DNA provided template using specific primers at the annealing temperature of 57°C in the presence of dNTP (0.1 mM), primers (0.5 *μ*M), and Taq DNA polymerase (Roche). 

### 2.5. Reverse Transcription and Real-Time Quantitative PCR (RT-qPCR)

Total RNA from Hepa 1.6 cells was extracted and purified using Qiagen RNeasy columns (Qiagen). One *μ*g of total RNA was used for reverse transcription with iScript Reverse Transcriptase (BioRad). PCR reactions were performed using the qPCR MasterMix Plus for SYBR Green I with fluorescein (Eurogentec). All PCR reactions were performed with MultiGuard Barrier Tips (Sorenson BioScience, Inc.) and an iCycler PCR machine (Bio-Rad Laboratories). Primers were designated to generate a PCR amplification product of 100–200 bp and were selected according to indication provided by the Primer 3 software (http://frodo.wi.mit.edu/primer3/). Sequences are available from S. Mandard on request. Specificity of the amplification was verified by melting curve analysis and evaluation of efficiency of PCR amplification. The “delta-delta Ct” quantification method was used and expression was related to the control gene 36B4, which did not change under any of the experimental conditions studied.

### 2.6. Transactivation Assays

COS-7 and HeLa cells were transfected with Exgen 500 (Euromedex) following manufacturer's protocol. 5 × 10^4^ cells/well were seeded in a 24-wells plate. Cells were transfected with a mixture of 1 *μ*g of plasmid DNA containing 30 ng of the reporter vector pCMV *β*-galactosidase (Clontech) together with equivalent molar amount of Luciferase (Luc) vectors (about 250 ng). Different amounts of expression vectors encoding for different nuclear receptors such as pSG5-mPPAR*α*, pSG5-mRXR*α*, and pCDNA3-hHNF4*α* were used. Corresponding empty vectors were used for control experiments. 4 h post transfection, the culture medium was replaced by 1 ml of complete medium with or without 10^−5^ M Wy (Alexis Biochemical). Luc and *β*-galactosidase activities were measured 48 h post transfection using the Promega Luc kit (Promega) and a standard assay. Chlorophenol red *β*-D-galactopyranoside was used as a substrate for *β*-galactosidase. For each condition, transfection assays were repeated four times. Reporter pGluc constructs was composed of a Luc expression vector containing the *β*-globin promoter upstream of the Luc coding sequence. TB PPRE3-pGluc was created by inserting synthetic double strand oligonucleotides between HindIII and BamHI sites of the pGluc vector (top strand _AGCT_^HindIII +935^ TGACCTGACCTCTGCTCGATAACCTTTTCCCTACTT^+970^, lower strand _GATC_^BamHI +970^ CTCTAAGTAGGGAAAAGGTTATCGGCAGAGGTCAGGTCA^+970^; ACOX-I PPRE, and MFP1-PPRE have been described previously [20, 21]. Hepa 1.6 cells (8 × 10^5^ in a 12-well plate) were transfected with Lipofectamine^TM^ 2000 (Invitrogen), H4IIEC3 cells (1 × 10^6^ in a 6-well plate) with FuGene HD (Roche) according to manufacturer's instructions.

### 2.7. Expression and Reporter Vectors

The expression vectors pSG5-mPPAR*α* and pSG5-mRXR*α* were a kind gift of Dr. Stephen Green (UK). The expression vectors encoding the mutated form of HNF4*α* (DN HNF4) was provided by Dr Todd Leff (Wayne State University School of Medicine, Detroit, Michigan, USA). DN HNF4 is a selective dominant negative mutant that forms defective heterodimers with WT HNF4*α* preventing DNA binding and transcriptional activation by HNF4*α*. DN HNF4 is unable to bind DNA on its own [22]. The mammalian expression vector encoding D126Y HNF4*α*2 was a kind gift of Dr. Bernard Laine (INSERM U459, Lille, France) [23].

### 2.8. Transient Transfections of Hepa 1.6 Cells with AMAXA Technology

Hepa 1.6 cells were routinely grown in DMEM medium supplemented with 10% Fetal Calf Serum. Hepa 1.6 cells (×10^7^) were resuspended in 100 *μ*l of the transfection solution provided with the transfection kit (Nucleofector kit V). Per transfection, 5 *μ*g of DNA were added to the cell suspension. The cell-DNA mix was transferred into the Amaxa transfection cuvette using the Amaxa minipipette. The cuvette was next placed into the Nucleofector and the program “T-028 High Efficiency” was selected. 500 *μ*l of warm media were added to the cuvette and transfected cells were transferred to the dish (25 cm^2^) with prewarmed media. 24 h post transfection, culture medium was changed. Total RNA was extracted 48 h post transfection. 

### 2.9. Gel shift and Supershift Assay

Nuclear extracts were prepared from transfected COS-7 cells (2 × 10^6^ cells per 100-mm dish, with 8 *μ*g of appropriate expression plasmid). Nuclei and nuclear extracts were prepared as previously indicated [24]. Protein concentrations were determined by the Bradford assay (BioRad). Oligonucleotides were end labelled with dCTP by using the Klenow fragment of DNA polymerase I. Nuclear extracts (0.5 *μ*g for hHNF4*α*-enriched extracts) were incubated for 1 h on ice in 20 *μ*l of the buffer (10 mM Hepes pH 7.9; 120 mM NaCl, 1 mM EDTA, 1 mM DTT, 7% (v/v) glycerol, 0.5 mM PMSF, 2 *μ*g each of leupeptin, aprotinin, pepstatin), 1 *μ*g of double-stranded poly (dIdC), and 35 fmol of radiolabelled rat PPRE3 oligonucleotide. Alternatively, a 1 to 100 fold molar excess of competitor oligonucleotides was added. The HNF4*α* antibody used for supershift was from Santa Cruz Biotechnology Inc. (HNF4*α* sc-8987x) and was added with the nuclear extract 30 minutes before adding the probe. Nucleoprotein complexes were resolved on a 6% (w/v) polyacrylamide gel in 1x TBE.

### 2.10. Coimmunoprecipitation (CoIP) Assays

For CoIP assays, nuclear extracts were adjusted to 25 mM HEPES (pH 7.9), 200 mM KCl, 1 mM EDTA, 0.5% NP-40, and 10% glycerol and incubated with 2 *μ*g of antibody at 4°C for 12 h. 50 *μ*l of protein G-Sepharose beads were washed twice with the same buffer before being incubated for 2 h with the upper mixture on a rotator. After a centrifugation step, pelleted beads were washed four times with the afore-mentioned buffer. After unbound proteins were washed away, bead pellets were finally resuspended in reducing loading buffer [25] and samples were boiled at 95–100°C for 3–5 minutes before being subjected to SDS-PAGE. The HNF4*α* (sc-6556), PPAR*α* (sc-9000x), RXR*α* (sc-774), as well as the secondary (donkey anti-goat, sc-2020) antibodies used were all purchased from Santa-Cruz Biotechnology Inc. 

### 2.11. DNA Affinity Precipitation Assay (DAPA)

DNA Affinity Precipitation Assay (DAPA) was performed as previously described [26]. Proteins were eluted in 15 *μ*l of SDS-PAGE loading buffer by heating for 5 min at 95°C. The streptavidin magnesphere paramagnetic beads were from Promega (Z5481). Similar antibodies as those used for coimmunoprecipitation assays were used except for the secondary antibody (goat antirabbit, sc-2004, Santa-Cruz Biotechnology Inc.).

### 2.12. Immunoblotting

Immunoblotting of the two peroxisomal 3-ketoacyl-CoA thiolases (PTL) was performed as previously described [27]. The rabbit polyclonal antibodies directed against both thiolase A and B proteins (PTL) was a gift from Dr. T. Hashimoto and Dr. N. Usuda (Shinshu University School of Medcine, Japan) and has been previously described [27]. Signals were detected with ECL-plus (Amersham Biosciences) according to the manufacturer's instructions.

### 2.13. Statistical Analyses

Unless indicated, the one-way ANOVA test was used to identify statistically significant differences. The cut off for statistical significance was set at a *P*-value of  .05 or below.

## 3. Results

### 3.1. Thb mRNA Levels Are Robustly Induced by the PPAR*α* Agonist Wy in Liver

**Figure 1:**
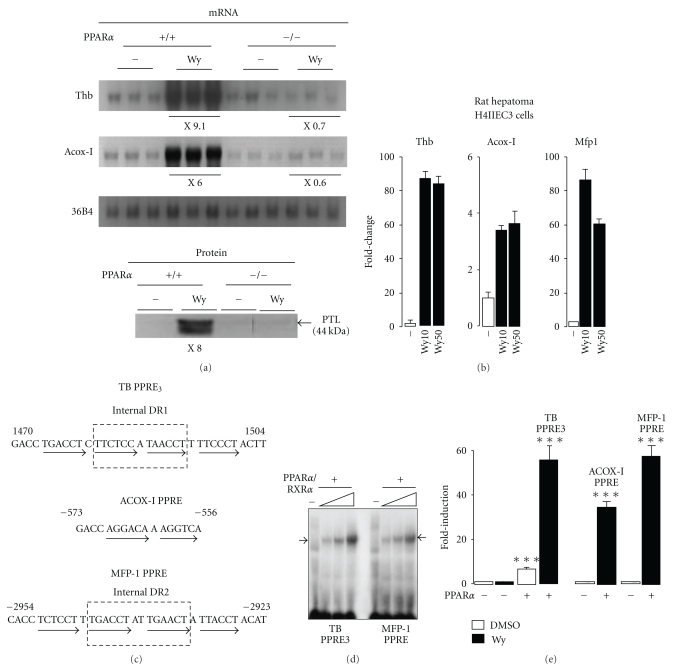
Wy induces hepatic *Thb* gene expression in a PPAR*α*-dependent manner through a novel PPRE. (a) Northern blot was performed with liver RNA from wild-type (*n* = 3) and PPAR*α* ^−/−^ mice (*n* = 3) fed Wy (30 mg kg^−1^·day^−1^) for 8 days. Autoradiographs were quantified and average fold changes indicated below the different blots. The signal for WT mice NOT fed Wy was arbitrarily set to 1. 36B4 mRNA levels were evaluated and used as internal control of loading. Western blot experiment was conducted with liver protein samples of the same animals using an antibody that recognizes the two peroxisomal 3-ketoacyl-CoA thiolases (PTL) identified in rodents [35]. (b) Rat hepatoma H4IIEC3 cells were treated with Wy (10 or 50 *μ*M, as indicated) for 48 h. Expression of Thiolase b (*Thb*), AcylCoA oxidase-1 (*Acox-1*), and Multifunctional protein-1 (*Mfp1*) was determined by quantitative RT-PCR analysis. Values are mean of three independent experiments ±SEM. Wy: Wy14,643. (c) The *Thb* gene contains a composite PPRE in intron 3. The nucleotide sequence of the different PPREs (TB PPRE3, ACOX-I PPRE, and MFP-1 PPRE) used in this study is shown. The nucleotide positions are given taking as +1 the transcription initiation site. The different arrows indicate half-site of a Direct Repeat (DR). (d) A double-strand oligonucleotide containing TB PPRE3 or MFP1-PPRE was incubated with increasing amounts of *in vitro* translated PPAR*α* and RXR*α* proteins. Binding complexes were separated by electrophoresis. (e) Transactivation assays were performed in COS-7 cells with a Luc reporter vector containing either a single copy of TB PPRE3 or a copy of ACOX-I PPRE or MFP-1 PPRE. These constructs were transfected together with an expression vector for mouse PPAR*α* (pSG5 mPPAR*α*) in presence or absence of Wy (10 *μ*M). DMSO was used as vehicle. Values are mean of four independent experiments ±SEM. ***Effect statistically significant compared to control (no PPAR*α* transfected, DMSO) with ***P < .001 with one-way Anova test. Errors bars represent SEM.

Using a cDNA probe recognizing both* Tha *and *Thb *genes, it was previously shown that *thiolase* (A + B) mRNA levels were induced by PPAR*α* agonists in a PPAR*α*-dependent manner [18]. To evaluate whether *Thb* only was sensitive to PPAR*α* agonists, northern blot analysis was performed using a specific nucleotide probe and hepatic RNA from WT and PPAR*α*-null mice treated or not with Wy14,643 (Wy), a potent and specific agonist of PPAR*α* (Figure 1(a)). 

Under basal conditions, *Thb* mRNA levels were similar in wild-type (WT) and PPAR*α*-deficient mice. The expression of the PPAR*α* target gene *Acox-I* and of *Thb* were induced in a PPAR*α*-dependent manner (Figure 1(a)). Of note, the augmentation of *Thb* mRNA levels upon Wy treatment was translated into higher peroxisomal 3-ketoacyl-CoA thiolases protein content (Figure 1(a)).

To further investigate whether the *in vitro* expression of *Thb* is also controlled by PPAR*α* in other species, rat hepatoma H4IIEC3 cells were treated with Wy. Using RT-qPCR, it was shown that *Thb* mRNA levels were robustly induced by activated PPAR*α* (Figure 1(b)). Given the dramatic activation of *Thb* expression by Wy in H4IIEC3 cells, more than a single PPRE in the rat *Thb* gene sequence may control its expression. Comparison with other established PPAR*α* target genes such as *Acox-I *and *Mfp-1* indicated that the gene expression profile of *Thb* was indeed closer to that of *Mfp-1* (one of the most responsive gene to PPAR*α* due to the presence of an atypical and composite PPRE in its gene sequence) than *Acox-I *(Figure 1(b)) [28, 29]. 

### 3.2. Identification of TB PPRE3 as a Novel Response Element for PPAR*α*

To find out for more functional DR1 PPREs, a comparative *in silico* analysis of the mouse and the rat *Thb *genes sequences from position −5600 bp upstream of the transcription start site to position +13000 bp downstream was performed with Nubiscan V2.0 software [30]. It revealed the presence of several conserved stretches of DNA sequences that harboured some previously characterized PPREs as well as a novel PPRE that we named TB PPRE3 [12, 15] (Figure 1(c)). Gel mobility shift assays were used to test whether TB PPRE3 was bound *in vitro* by translated PPAR*α*/RXR*α* proteins. In agreement with data previously reported by others, we found a retarded PPAR*α*/RXR*α* complex on MFP-1 PPRE but also on TB PPRE3, further implying direct regulation of *Thb* by PPAR*α* through TB PPRE3 [28] (Figure 1(d)). 

To verify whether PPAR*α* can transactivate the *Thb* gene through TB PPRE3, transactivation assays were performed in COS-7 cells with a reporter vector containing a single copy of TB PPRE3. Luc activity of the reporter vector containing TB PPRE3 responded significantly to PPAR*α* overexpression in the absence of exogenous agonist (Figure 1(e)). Since the apparent large responsiveness of PPAR*α* in absence of exogenous ligand may reflect the high constitutive activity mediated by the ligand-independent AF1 domain, we examined the behaviour of N-terminally deleted (ΔAF1) PPAR*α* construct that lacks the AF1 in transactivation assays. It was found that ΔAF1 PPAR*α* behaved similarly to WT PPAR*α*, suggesting the presence of natural PPAR*α* agonists in the culture medium (data not shown). As expected, Luc activity was significantly enhanced after Wy treatment. It is important to note that the MFP-1 PPRE is composed of two DR1 elements separated by two base pairs, thereby forming an internal DR2 element (Figure 1(c)) [28, 29]. With the exception of a single-base pair separating two putative DR1, the overall structure of TB PPRE3 was quite similar to that of MFP-1 PPRE. Yet, it is also worth noting that the nucleotide sequence composing the four different half-sites of TB PPRE3 significantly differs from that of MFP-1 PPRE. Of interest and compared to ACOX-I PPRE, the fold induction for TB PPRE3 and MFP-1 PPRE were quite similar as a probable consequence of their unusual and composite structure (Figures 1(c) and 1(e)). Overall, TB PPRE3 is a potent response element for PPAR*α* and is likely critical for PPAR*α*-dependent activation of *Thb *gene transcription. 

### 3.3. The Nuclear Orphan Receptor HNF4*α* Controls Thb Gene Expression in Liver and in Hepatoma Hepa 1.6 Cells

**Figure 2:**
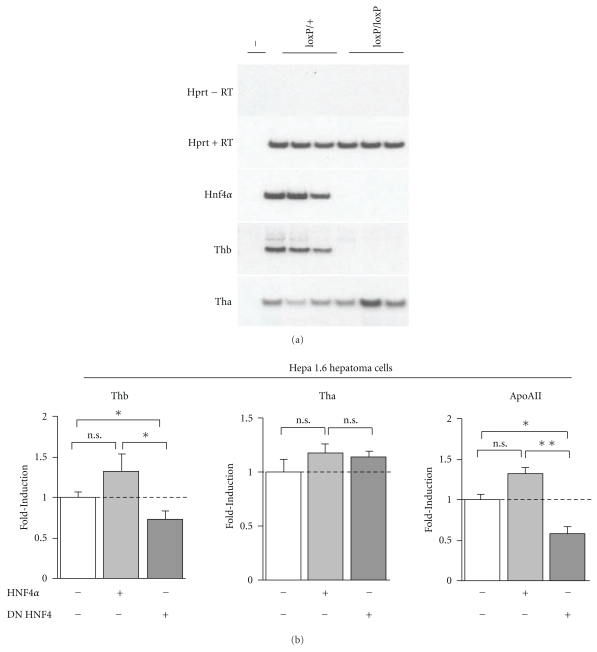
HNF4*α* is a master gene for the hepatic expression of *Thb*. (a) Expression of hepatic *Thb* in HNF4*α* null (*Hnf4* ^loxP/loxP^ *Alfp.cre*; loxP/loxP) and control (*Hnf4* ^loxP/+^ *Alfp.cre*; loxP/+) mouse embryos was determined by semiquantitative RT-PCR analysis (*n* = 3 per group). *Hprt* mRNA livers were evaluated and used as internal standard of loading. *Tha*: thiolase A; *Thb*: thiolase B; *Hnf4α*: Hepatocyte Nuclear Factor-4 alpha; *Hprt*: Hypoxanthine-guanine PhosphoRibosyl Transferase. (b) Mouse Hepa 1.6 hepatoma cells were transfected with expression vectors for WT HNF4*α* or dominant negative form of HNF4*α* (DN HNF4). Expression of *Thb*, *Tha*, and apolipoprotein AII (*ApoA-II*) was determined by quantitative RT-PCR. Crude results were standardized against 36B4 mRNA levels. Levels of gene expression in Hepa 1.6 cells transfected with empty pcDNA3.1 vector serve as reference point and are given the arbitrary value of 1.0. Values are mean of three independent experiments ±SEM. Apolipoprotein AII (ApoAII) gene expression was used as a positive control of experiment. Significantly different compared to control (transfection with empty pcDNA3.1 vector) with *P < .05 and **P < .01 by one-way ANOVA test. n.s.: no statistically different compared to control.

The liver Hepatocyte Nuclear Factor-4*α* (HNF4*α*, NR2A1) plays an important role in the transcriptional regulation of genes involved in different metabolic pathways, including fatty acid, amino acid, glucose, and cholesterol metabolism. Although limited, there is some overlap in the identity of some of the HNF4*α* and PPAR*α* regulated genes [6, 31, 32]. In view of this, HNF4*α* might be hypothesized to directly control *Thb* gene expression in liver. Because the complete deletion of *Hnf4α* is lethal in mice, we sought to check whether deletion of *Hnf4α* had any impact on liver *Tha* and *Thb* basal expression using samples from *Hnf4α* ^−/−^ 18.5 days old embryos (Figure 2(a)) [16, 19]. 

While mRNA levels of *Tha* were not affected, *Thb* mRNA levels were markedly decreased in liver of *Hnf4* ^loxP/loxP^ *Alfp.cre* embryos supporting a critical role for HNF4*α* in regulating the basal expression of* Thb *during development of hepatocytes (Figure 2(a)). It may also be hypothesized that the decrease in* Thb* mRNA levels observed in liver of the *Hnf4* ^loxP/loxP^ *Alfp.cre* embryos could be the consequence of metabolic perturbations due to the lack of HNF4*α*. To exclude this possibility, and since we have no way to pharmacologically activate HNF4*α in vivo*, we studied the expression of *Thb* in mouse Hepa 1.6 hepatocytes that were efficiently transfected (using The Amaxa Nucleofector technology) with either a WT form or with the potent dominant-negative form of HNF4*α* (DN HNF4) previously characterized [22]. DN HNF4 is a selective dominant negative mutant that contains a defective DNA-binding domain. Hence, DN HNF4 forms defective heterodimers with WT HNF4*α* thereby preventing DNA binding and subsequent transcriptional activation by HNF4*α*. Compared to COS-7 and HeLa cells, mouse Hepa 1.6 and rat H4IIEC3 hepatoma cells express large amount of endogenous WT HNF4*α* (data not shown). Ectopic expression of WT HNF4*α* in Hepa 1.6 hepatocytes had a minor impact on *Thb *and* Tha *mRNA levels, as shown by RT-qPCR (Figure 2(b)). A similar picture was also observed for *ApoAII, *a previously characterized HNF4*α* target gene [33]. However, forced expression of DN HNF4 decreased *Thb *as well as* ApoAII *mRNA levels by 50% while *Tha* mRNA remained unchanged, confirming that HNF4*α* controls the basal *Thb* gene expression. Together, our data indicate that HNF4*α* is a novel regulator of *Thb* expression in cell lines of hepatic origin. 

### 3.4. HNF4*α* Binds to TB PPRE3

**Figure 3:**
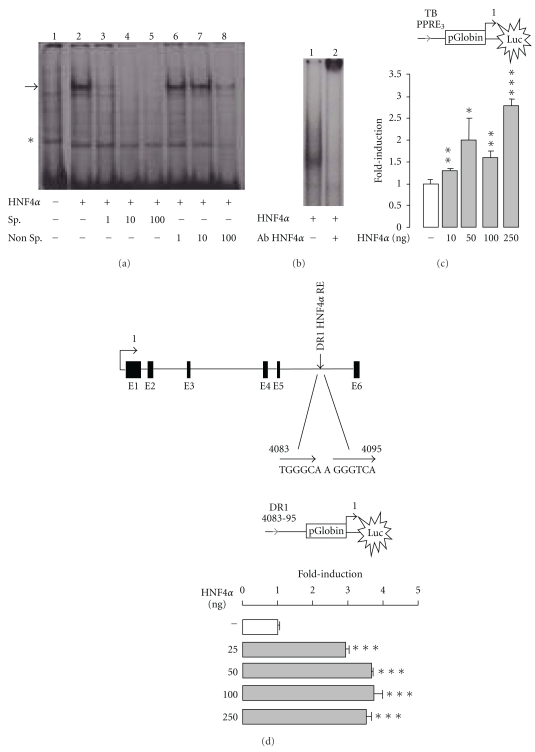
TB PPRE3 is a novel binding site for the nuclear receptor HNF4*α.* (a) Binding of HNF4*α* to native radiolabelled (^32^P) TB PPRE3 was determined by gel shift assay. A double strand oligonucleotide containing (^32^P) TB PPRE3 was incubated with lysates of transfected (by the expression vector HNF4*α*) COS-7 cells. Fold excess of specific (Sp.) cold probe (PPRE of the peroxisomal ACOX-I gene) was used for data shown lanes 3, 4 and 5. Nonspecific (Non Sp.) cold probe (Sp1) was used for data shown lanes 6, 7 and 8. Binding complexes were resolved on a 6% non-denaturing polyacrylamide gel. The arrow indicates the specific binding of HNF4*α*. The star indicates nonspecific binding. (b) Supershift assay (lane 2) was performed with an antibody directed against HNF4*α*. (c) COS-7 cells were transfected with increasing amounts (0 to 250 ng) of expression vectors encoding WT HNF4*α* with a Luc reporter vector containing TB PPRE3. (d) COS-7 cells were transfected with increasing amounts (0 to 250 ng) of expression vectors encoding wild-type HNF4*α* with a Luc reporter vector containing one copy of the DR1 localized in intron 5 of the mouse version of *Thb* (+4083 +4095 bp). Values are mean of three independent experiments ±SEM. Significantly different compared to control (transfection with empty pcDNA3.1 vector) with *P < .05 by one-way ANOVA test.

Given that HNF4*α* is critical for the basal expression of *Thb* and that HNF4*α* recognizes DR1 sequences, we examined whether HNF4*α* may bind to TB PPRE3 [34]. Using TB PPRE3 as a probe together with HNF4*α*-enriched nuclear extracts from HNF4*α*-transfected COS-7 cells, we performed electrophoresis mobility shift assays. A complex was seen when enriched HNF4*α* nuclear extracts were used (Figure 3(a), lane 2). 

This complex mainly contained HNF4*α* since it was absent in untransfected COS-7 cells (lane 1) and it disappeared upon the addition of an excess amount of the unlabelled ACOX-I PPRE consensus oligonucleotide (lane 3 to lane 5), previously shown to be efficiently bound by HNF4*α*. An excess amount of cold nonspecific Sp1 binding site oligonucleotide did not decrease HNF4*α* binding to PPRE3 (lane 6 to lane 8). Addition of HNF4*α* specific antibody with the nuclear HNF4*α* enriched extracts supershifted the complex (Figure 3(b), lane 2). Together, our data demonstrate the *in vitro* binding of HNF4*α* to TB PPRE3.

To assess whether TB PPRE3 is able to mediate HNF4*α*-dependent transactivation, transient transfections were performed with HNF4*α* and reporter vectors containing a single copy of TB PPRE3 sequence. While significant, HNF4*α* only modestly modulated transcription *via *TB PPRE3 in COS-7 cells (Figure 3(c)) indicating that TB PPRE3 behaves poorly as a HNF4*α* response element in classical transactivation assays and suggesting that binding of HNF4*α* to TB PPRE3 is not necessarily translated into a massive transcriptional activation. Additional transactivation assays performed with 5.65 kb of the mouse *Thb* gene promoter sequence also failed to demonstrate HNF4*α*-dependent promoter activation (data not shown). We concluded that the critical response element(s) for HNF4*α*, if any, was likely located elsewhere. 

In light of this consideration, Bolotin et al. recently reported on an integrated approach for the identification of human HNF4*α* target genes using protein binding microarrays [35]. This strategy allowed the discovery of a DNA sequence bound by HNF4*α* in the human version of the peroxisomal 3-ketoacylCoA thiolase (also known as 3-acetyl-CoA acetyltransferase-1, ACAA1). Because we found a similar sequence in intron 5 (DR1int5) of the mouse *Thb* gene, transactivation assays were performed in COS-7 cells using a copy of DR1int5 cloned in front of the luciferase reporter gene (Figure 3(d)). Transfection with HNF4*α* enhanced reporter activity to about 3-fold, suggesting that DR1int5 is partly involved in the regulation of *Thb* by HNF4*α*. 

### 3.5. HNF4*α* Enhances the PPAR*α*-Mediated Activation of Transcription from TB PPRE3

**Figure 4:**
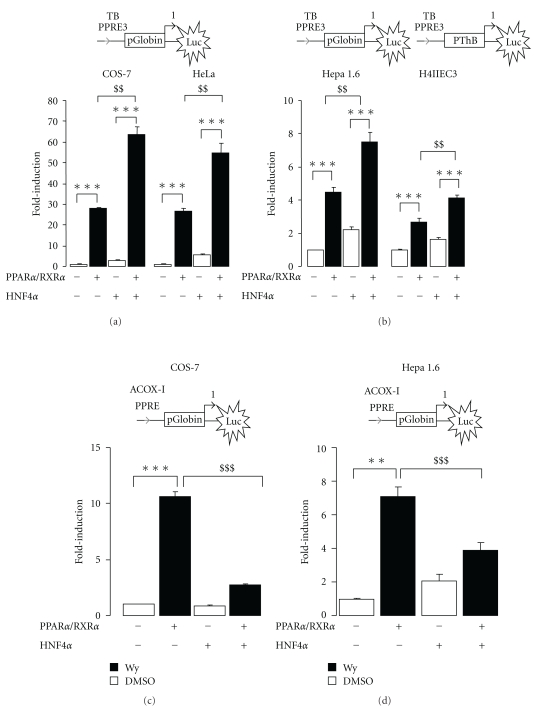
PPAR*α* transactivation from TB PPRE3 is markedly enhanced by HNF4*α*. (a) Transactivation assays were performed in COS-7 and HeLa cells (b) or in Hepa 1.6 and H4IIEC3 cells with a Luc reporter vector containing isolated rTB PPRE3. These constructs were transfected together with expression vectors for both mouse PPAR*α* (pSG5-mPPAR*α*) and RXR*α* (pSG5-mRXR*α*) in absence (white bars) or presence (black bars) of Wy (10 *μ*M). Cotransfection with pcDNA3.1 WT hHNF4*α* was performed as indicated. Note that the minimal promoter of the *thiolase B* gene was used instead of the globin gene promoter which was inactive in H4IIEC3 cells. (c) Transactivation assays were performed in COS-7 or Hepa 1.6 cells with a Luc reporter vector containing isolated ACOX-I PPRE. Normalized luciferase activity of each construct in the absence of PPAR*α* and ligand was set at 1. Values are mean of four independent experiments ±SEM. DMSO was used as vehicle. Significantly different compared to control (transfection with a combination of empty pcDNA3.1 and pSG5 vectors) with **P < .01 and ***P < .001 by one-way ANOVA test. Significantly different between PPAR*α*/RXR*α* and PPAR*α*/RXR*α* + HNF4*α* with ^$$^ *P* < .01 and ^$$$^ *P* < .001 by one-way ANOVA test.

It is widely documented that HNF4*α* and PPAR*α* share some similar binding motifs leading to competition between these two receptors. To check whether the same holds true for TB PPRE3, both receptors were cotransfected either in nonhepatic (COS-7, HeLa) or hepatic cells (Hepa 1.6, H4IIEC3). Whatever the cell line used, PPAR*α*/RXR*α* heterodimer increased reporter activity which was even further enhanced by cotransfection of HNF4*α* (Figures 4(a) and 4(b)).

As previously reported by others, cotransfection with HNF4*α* strongly suppressed PPAR*α*/RXR*α* increased reporter activity when Luc activity was driven by the ACOX-I PPRE motif (Figure 4(c)). Hence, we concluded that HNF4*α* promotes transactivation mediated by PPAR*α*/RXR*α* from TB PPRE3 through a molecular mechanism that remains to be determined.

### 3.6. Binding of HNF4*α* to TB PPRE3 is Dispensable for the Cooperation with PPAR*α*

**Figure 5:**
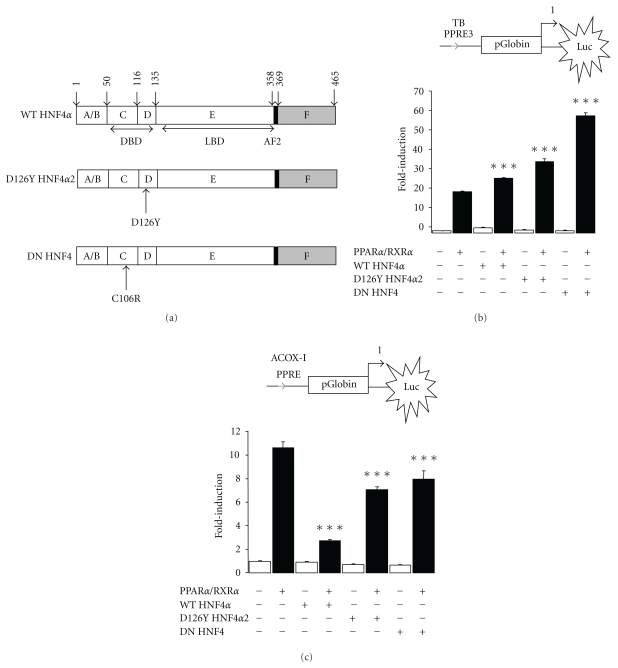
Binding of HNF4*α* to TB PPRE3 is dispensable for the cooperation with PPAR*α*/RXR*α*. (a) Positions of mutations used in this study. A scheme of HNF4*α* structure with the various domains is given: DBD, DNA-binding domain; LBD, ligand-binding domain; AF2, activation function 2 module. (b) Transactivation assays were performed in COS-7 cells with a Luc reporter vector containing isolated TB PPRE3 (b) or mouse ACOX-I PPRE (c). These constructs were cotransfected with expression vectors for both mouse PPAR*α* (pSG5-mPPAR*α*) and RXR*α* (pSG5-mRXR*α*) together with an expression vector encoding either wild-type HNF4*α* (HNF4*α*2 WT), a first (D126Y HNF4*α*2) or a second deficient form (DN HNF4) of HNF4*α* for DNA binding in absence (white bars) or presence (black bars) of Wy (10 *μ*M). Values are mean of three independent experiments ±SEM. DMSO was used as vehicle. Significantly different between PPAR*α*/RXR*α* and PPAR*α*/RXR*α* + HNF4*α* with **P < .01 and ***P < .001 by one-way ANOVA test.

Inasmuch HNF4*α* positively influenced the ability of PPAR*α* to transactivate the TB PPRE3, it may suggest a possible cooperation between both receptors. To test whether this synergistic effect by HNF4*α* depends on its capacity to bind to TB PPRE3, transactivation assays were conducted in COS-7 cells using two different mutant forms of HNF4*α* for which DNA binding is either limited (−75%, as a consequence of a point mutation within its DNA binding domain, D126Y HNF4*α*2) or abolished (DN HNF4) while their stability remains unaffected (Figure 5(a)) [22, 23]. 

While the transactivation potential of D126Y HNF4*α*2 is reduced, dimerization of the receptor was not reported to be affected. Similar to WT HNF4*α*, over-expression of either D126Y HNF4*α*2 or DN HNF4 failed to transactivate the native TB PPRE3 (Figure 5(b)). Noteworthy, both D126Y HNF4*α*2 and DN HNF4 potentiated further than WT HNF4*α* the transactivation by PPAR*α* from TB PPRE3. Together, we conclude that the cooperation between HNF4*α* and PPAR*α* was not dependent on the physical binding of HNF4*α* to TB PPRE3.

To finally check whether the cooperative effect between PPAR*α* and HNF4*α* depends on the DNA nucleotide sequence itself, we performed transactivation assays using the DR-1 PPRE of the Acyl-CoA Oxidase I (ACOX-I PPRE) (Figure 5(c)). In contrast to TB PPRE3, overexpression of WT HNF4*α* potently decreased Luc activity by the activated PPAR*α*/RXR*α* heterodimer. Transfecting expression vectors encoding the two mutant forms of HNF4*α* led to a comparatively moderate decrease in Luc activity. Therefore, we hypothesized that the competition between PPAR*α* and HNF4*α* was reduced. The two mutants were unable to drive Luc activity to a level higher than the PPAR*α*/RXR*α* heterodimer alone, confirming the lack of cooperation with HNF4*α*. Overall, our data suggest that the cooperation between HNF4*α* and PPAR*α* receptors may depend on both the structure and the nucleotide sequence of the DNA response element.

### 3.7. HNF4*α* Does Not Favour the Binding of PPAR*α*/RXR*α* to TB PPRE3

**Figure 6:**
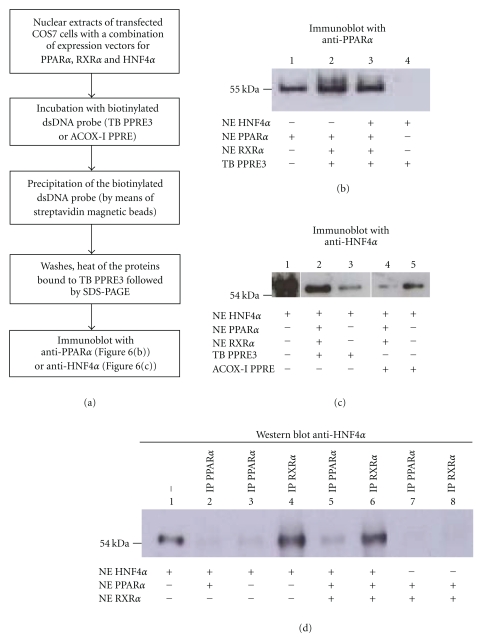
Binding of PPAR*α*/RXR*α* to TB PPRE3 is not enhanced by HNF4*α*. (a) Schematic outline of the DAPA procedure. (b) DAPA was performed with nuclear extracts of transfected COS-7 cells (with either HNF4*α*, PPAR*α*, or RXR*α*) using the biotinylated TB PPRE3 oligonucleotides. Complexed proteins were resolved by SDS-PAGE and revealed by Western blot using an anti-PPAR*α* (Figure 6(b)) or anti-HNF4*α* antibody (Figure 6(c)). (d) Coimmunoprecipitation of RXR*α* and HNF4*α*. Nuclear extracts from transfected COS-7 cells were immunoprecipitated (IP) with PPAR*α* or RXR*α* antibodies, as indicated. The total plasmid amount was adjusted with pCDNA3.1 parent vector to 8 *μ*g for each 100-mm transfection with Exgen 500. Cells were incubated in DMEM 10% FCS with 10 *μ*M Wy for 48 h. The presence of HNF4*α* protein in the immunopurified material (100 *μ*g of nuclear protein) was detected by Western blot assay using anti-HNF4*α* antibody. NE: Nuclear Extracts. Lane 1: Only 4 *μ*g of nuclear protein were loaded.

The molecular mechanisms by which the stimulatory effect of HNF4*α* on PPAR*α*/RXR*α*-mediated activation of transcription *via* TB PPRE3 does not require the binding of HNF4*α* to DNA but at the present time, this mechanism remains unclear. We, therefore, questioned whether HNF4*α* could facilitate the binding of the PPAR*α*/RXR*α* heterodimer to TB PPRE3, *via* a possible protein-protein interaction. We used DNA Affinity Precipitation Assay (DAPA) to assess complex formation on TB PPRE3 with PPAR*α*, RXR*α* and HNF4*α* proteins coming from nuclear extracts of transfected COS-7 cells (Figure 6(a)). 

Ectopic expression of PPAR*α* in COS-7 cells led to the production of the PPAR*α* protein (Figure 6(b), lane 1) and overexpressed HNF4*α* was not detected by the PPAR*α* antibody (Figure 6(b), lane 4). Consistent with our previous findings (Figure 1(d)), we confirmed that PPAR*α* was bound to TB PPRE3 (Figure 6(b), lane 2). When PPAR*α*, RXR*α*, and HNF4*α* proteins were incubated altogether with TB PPRE3, the signal was similar indicating that HNF4*α* does not favor the binding of PPAR*α*/RXR*α* to TB PPRE3. 

We, therefore, concluded that the enhancement of the PPAR*α*/RXR*α* mediated-activation of transcription from TB PPRE3 by HNF4*α* was likely not due to DNA binding stabilization of the PPAR*α*/RXR*α* heterodimer.

To go a step further, immunorevelation of the complexes was also performed with a HNF4*α* antibody that recognized ectopic WT HNF4*α* (Figure 6(c), lane 1) but not PPAR*α* or RXR*α* (data not shown). As previously shown in this study, HNF4*α* was either physically bound and/or was part of a complex bound to TB PPRE3 (Figure 6(c), lane 3). Intriguingly, when PPAR*α*, RXR*α* and HNF4*α* proteins were incubated altogether (Figure 6(c), lane 2), the signal for HNF4*α* was stronger supporting the notion that PPAR*α*/RXR*α* may favour the binding and/or the recruitment of HNF4*α* to TB PPRE3. In contrast to TB PPRE3 and in support of transactivation assays from others, competition between PPAR*α*/RXR*α* and HNF4*α* for binding to ACOX-I PPRE was found, validating our experimental system (Figure 6(c), compare lane 4 and lane 5) [36].

### 3.8. Physical Interaction between RXR*α* and HNF4*α*

The detection of increased HNF4*α* protein on the TB PPRE3 in the presence of PPAR*α* and RXR*α* may result from physical interaction between PPAR*α*/RXR*α* and HNF4*α*. This hypothesis merits further investigation especially because we found that DN HNF4*α* (deficient for DNA binding) was able to further enhance the PPAR*α*/RXR*α* transactivation from TB PPRE3, possibly *via* protein-protein interactions (Figure 5(b)). Therefore, the interaction of PPAR*α*, RXR*α* or PPAR*α*/RXR*α* with HNF4*α* was assessed in solution in the absence of DNA using coimmunoprecipitation (CoIP) assays. Nuclear protein extracts from transfected COS-7 cells (see details, Figure 6(d)) were first immunoprecipitated with a polyclonal PPAR*α* antibody that mapped the N-terminus part of the protein, before being analyzed by Western blotting with a polyclonal HNF4*α* antibody (Figure 6(d), lanes 2, 3, 5, and 7). In agreement with the lack of endogenous HNF4*α* in COS-7 cells, no signal was observed in absence of ectopic HNF4*α* (Figure 6(d), lane 7). Transient transfection of HNF4*α* led to the appearance of very faint bands (Figure 6(d), lanes 2 and 3) possibly indicating a weak interaction between PPAR*α* and HNF4*α*. When PPAR*α*, RXR*α* and HNF4*α* proteins were present altogether (Figure 6(d), lane 5), a little signal was still observed suggesting that PPAR*α* either directly or *via* another protein partner such as RXR*α*, may interact with HNF4*α*. Alternatively, we cannot exclude the possibility that the interaction between HNF4*α* and PPAR*α*/RXR*α* may partly mask the epitope recognized by the PPAR*α* antibody. 

To go a step further in our study, CoIP assays were also performed using a polyclonal RXR*α* (the obligate PPAR*α* partner) for the immunoprecipitation step. Remarkably, it was found that RXR*α* massively interacts with HNF4*α* in solution (Figure 6(d), lane 6). It is worth noting that when the immunoprecipitation step was performed with the HNF4*α* antibody and the Western blotting with the RXR*α* antibody respectively, we and others failed to observe any signals [37] (data not shown). This might be the consequence of an epitope mapping at the C-terminus of HNF4*α*, a region critical for protein-protein interaction. Therefore, the epitope may be not accessible to the antibody used.

Because the endogenous PPAR*α* expression is barely detected in COS-7 cells (data not shown) and since the signal observed in absence of ectopic PPAR*α* was similar (Figure 6(d), compare lane 4 and 6), it can be concluded that HNF4*α* interacts with RXR*α* irrespective of the presence of PPAR*α*. The signals observed in lanes 4 and 6 are in concordance with the apparent molecular weight obtained with the ectopic transfected WT HNF4*α* (Figure 6(d), lane 1). 

Overall, our results point towards a physical interaction in solution between HNF4*α* and RXR*α*. 

### 3.9. HNF4*α* is Involved in the PPAR*α*-Regulated Induction of Thb in the Liver

**Figure 7:**
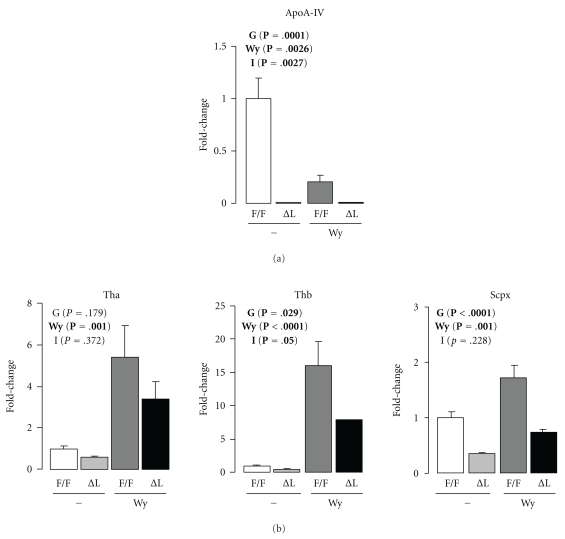
The PPAR*α*-regulated induction of *Thb* is potentiated by HNF4*α* in the liver. Hepatic mRNA levels in the liver-specifically HNF4*α*-disrupted (HNF4*α* ∆L) and HNF4*α* F/F-fed control or Wy14,643-containing diet (0.1% w/w) for five days. Total RNA extracted from livers of these mice were subjected to real-time PCR analysis. The expression signals from the WT mice that did not receive Wy were arbitrarily set at 1. The results are shown as a relative expression to *β*-actin mRNA levels as normalization control. *Error bars* represent standard error (SE) and data are expressed as the mean ± S.E. (*n* = 4 for each condition). Significant effects were calculated using two-way ANOVA test for the genotype (G), Wy14,643 (Wy) and the interaction between both parameters (I). Results are indicated at the top of each figure. In bold, parameters that are under the cutoff for statistical significance (*P* value of .05 or below). Wy: Wy14,643.

In order to check whether our *in vitro* findings are translated *in vivo *and because a dichotomy may exist in the function of HNF4*α* in adult and fetal liver, we next explored the impact of *Hnf4α* deletion on basal and Wy-induced expression of *Thb*, *Tha*, and SCPx/SCP2 thiolase in adult mice with conditional hepatic disruption of HNF4*α* (∆L HNF4*α*) [17]. Our experimental conditions reproduced the classical pattern of *ApoAIV* expression associated with the selective *Hnf4α* deficiency in the liver (Figure 7). When compared to WT mice, the expression of *Thb* and *Scpx* was significantly lower in ∆L HNF4*α* mice (*P* = .029 and *P* < .0001 for *Thb* and *Scpx*, resp.). *Tha *expression was not affected by hepatic Hnf4*α* deletion (*P* = .179). This piece of data supports our previous finding that *Tha* is not a HNF4*α* regulated gene. Furthermore, levels of mRNA for *Thb*, *Tha,* and *Scpx* were all significantly induced by Wy (*P* = .001, *P* < .0001 and *P* = .001 for *Thb*, *Tha*, and *Scpx*, resp.). Of note, the effect of HNF4*α* was Wy-sensitive (Wy∗genotype interaction, I: *P* = .05) only for *Thb*. While the expression of *Tha* and *Scpx *tended to be lower in ∆L HNF4*α* mice fed Wy (*P* = .372 and .228 for *Tha *and *Scpx*, resp.), it was not significant. Together, our data support the involvement of HNF4*α* in the PPAR*α*-regulated induction of *Thb in vivo.*

## 4. Discussion

This study contributes to the understanding of the regulation of the *Thb* gene by the nuclear receptors PPAR*α* and HNF4*α*. Under basal conditions, hepatic *Thb* mRNA levels were not affected by PPAR*α* deletion, similar to other direct PPAR*α* target genes such as the G0/G1 switch gene 2 or the gene encoding the soluble Interleukin-1 Receptor antagonist [38, 39]. This is not surprising since either a physiological stimulus such as fasting or a chronic stimulus like high fat diet is required for the activation of PPAR*α*-dependent signalling system in liver [40, 41]. Disruption of PPAR*α* completely abolished Wy-mediated induction of *Thb* mRNA levels. Because the induction of *Thb* gene expression by PPAR*α* agonists was robust, we hypothesized the presence of more than a single functional PPRE within the promoter and/or *Thb* gene sequence. In line with this, we previously reported on the characterization of TB PPRE2 by which PPAR*α* can transactivate [12]. This followup study now brings evidence that the molecular regulation of *Thb* by PPAR*α* in liver is more complex than previously expected since it also depends on a novel cis-acting element that we named TB PPRE3, an atypical PPRE composed of two sequential DR1 sequences separated by one nucleotide thereby forming an internal DR1 element. 

The presence of more than a single PPRE in close proximity (so-called PPRE clusters) has been proposed for the mouse and human version of the *Fiaf/Angptl4* and *Ucp3 *genes as well as for the mouse catalase and rat Cyp4a1 genes [9, 42, 43]. Four adjacent PPREs are present in intron 3 of the highly PPAR sensitive target *Fiaf/Angptl4 *but only a single PPRE is functional [44]. It can be argued that in contrast to *Thb*, no overlapping was reported for these four PPREs rending only speculative a potential comparison between the two situations. While the presence of more than a single PPRE in a genomic sequence is not unique, overlapping of PPREs appears to be a rare occurrence. Wen et al. recently reported that the mouse *Octn2* gene contains three overlapping PPREs in intron 1, yet only a single PPRE was predominant [34]. Similar to *Thb*, the presence of three PPREs in the genomic sequence of *Octn2* is likely responsible for its massive transcriptional response to activated PPAR*α.*

With respect to other peroxisomal genes, the *SCPx/SCP2* thiolase gene was previously identified as a target of PPAR*α* with two separated DR1 PPRE motifs localized in the promoter region [45]. It is also worth noting that *Mfp1* displays a PPRE composed of four consensus hexameric TGACCT half-sites in an arrangement of two DR1 elements separated by two base pairs, thereby also forming an internal DR2 element [28, 29]. Similar to *Mfp1*, *Thb *appears to be the second peroxisomal oxidative gene that displays this particular feature. 

Although the presence of multiple PPREs may be responsible for the large responsiveness of *Thb* mRNA levels to activated PPAR*α*, alternative explanations are also considered. In this respect, previous data have shown that *Thb* mRNA was positively regulated by the liver X receptor alpha [46]. Given that transcription of the liver X receptor alpha gene has been proposed to be dependent on PPAR*α*, it can be concluded that the PPAR*α* signalling pathway modulates liver *Thb* expression through distinct but complementary mechanisms [47].

Furthermore, flanking the internal DR1 element of TB PPRE3 are two half-sites that have been shown to enhance and stabilize the formation of dimeric complexes made by type II nuclear receptors such as RXR and the thyroid hormone receptor [48]. Therefore, one can make the attractive hypothesis that such a mechanism also takes place as far as the PPAR/RXR heterodimer is concerned. It could then partly support the large response of the reporter vector to PPAR*α* agonists in transactivation assays. Moreover, using electrophoretic mobility shift assays, others have previously classified the binding efficiency of PPAR*α* to MFP-1 PPRE as high and transactivation assays further supported the functional relevance of this classification [49]. Given that the structure of TB PPRE3 is close to that of the MFP-1 PPRE, the presence of the TB PPRE3 in the genomic DNA sequence of *Thb* may be enough to explain the large responsiveness of *Thb* mRNA levels to PPAR*α* agonists.

In addition to PPAR*α*, the nuclear receptor HNF4*α* is abundantly expressed in liver and shares with PPAR*α* some similar DNA-binding properties [50]. Because several DR1 motifs (potential candidates for HNF4*α* binding) have been identified in the sequence of *Thb*, regulation of *Thb *by HNF4*α* was studied using RNA from HNF4*α* null embryos and HNF4∆L adult mice. It was found that hepatic *Thb *mRNA levels were dependent on HNF4*α* because it was either completely absent (embryo) or strongly reduced (adult) in liver upon HNF4*α* ablation. Yet, HNF4*α* transactivations from TB PPRE3 or DR1int5 were rather disappointing. At this point of investigation, our data of transactivation assays about HNF4*α* and TB PPRE3 or DR1int5 are at odds with the clear cut picture of *Thb *gene expression in the liver of HNF4*α*-deficient embryos/mice. A plausible explanation is that another critical HNF4*α*-response elements might be located elsewhere in the genomic DNA. Theoretically, it is also possible that taken individually, the DR1s tested are poor HNF4*α*-response elements while together in the genomic context of the *Thb* gene sequence, these distal elements simultaneously act to trigger a massive transcriptional response. Alternatively, we cannot rule out that a critical response element for HNF*4α* is located very far from the initiation start site of *Thb* in another part of the chromosome that would bend to specifically interact with the genomic sequence of *Thb*. Such a regulation has been recently described for the regulation of *Ucp2* and *Ucp3* by the nuclear receptor PPAR*γ* [51]. 

Moreover, it is difficult to estimate to what extent PPAR*α* might be involved in the lack of *Thb *observed *in vivo* in HNF4∆L mice. Different studies have provided evidence that the steady-state levels of PPAR*α* mRNA were decreased in the liver of HNF4∆L adult mice [17, 52]. Ongoing investigations by DNA ChIP also showed that the PPAR*α* promoter was physically bound by HNF4*α* classifying PPAR*α* as a novel and direct target of HNF4*α* [52]. Interestingly, binding of PPAR*α* to the HNF4*α* promoter/enhancer was also reported [52]. Therefore, the authors concluded the existence of combinatorial regulation of the expression of *Pparα* and *HNF4α*, acting in a coordinated fashion on their downstream targets genes. Because basal *Thb *mRNA levels in the liver of PPAR*α* null and WT mice was similar under regular conditions, a critical role for PPAR*α* in *Thb* regulation is unlikely in HNF4∆L mice. 

Besides its classification as a strong constitutive transcriptional activator, it was previously demonstrated that HNF4*α* can form a stable affinity complex with other transcription factors such as SHP, SREBP-1c, SREBP-2, and HNF1*α* leading to the modulation of the expression of some of their respective target genes [53, 54]. These data raise an important conceptual question about the comparison between HNF4*α* as a conventional nuclear receptor/transcription factor and coactivator. Our data are consistent with the explanation that HNF4*α* interacts with RXR*α* in solution (Figure 6(d)) and in the presence of DNA (Figure 6(c)) without disrupting the binding of the liganded PPAR*α*/RXR*α* heterodimer (Figure 6(b)). 

While further studies are necessary to determine the stoichiometry of this interaction, it is worth underlining that HNF4*α* potentiated the transactivation from TB PPRE3 by liganded PPAR*α*/RXR*α* in four different cell lines ruling out the hypothesis of an artefact due to the use of a particular cell line*.* Our data are reminiscent of those obtained by Winrow et al. who previously reported on the functional cooperation between PPAR*α* and HNF4*α* in the induction of Luc activity from the MFP-1 PPRE [36]. As previously reported by others, HNF4*α* decreased transcriptional activation of ACOX-I PPRE by PPAR*α*/RXR*α* [36, 55]. Importantly, our current finding that HNF4*α* is involved in the PPAR*α*-induced expression of *Thb* in liver in mice indicates that our data of transactivation assays observed in COS-7 cells are translated *in vivo*. 

The lingering question arises why HNF4*α* would enhance transactivation from TB PPRE3 and MFP-1 PPRE while decrease that from ACOX-I PPRE? The molecular mechanism by which HNF4*α* influences transactivation by PPAR*α*/RXR*α* from TB PPRE3 likely involves interaction between HNF4*α* and RXR*α*. Furthermore, the specific association of coactivators/corepressors with PPAR*α*/RXR*α* or the configuration of the chromatin might strengthen or weaken the stability of PPAR*α*/RXR*α* to the different PPREs rendering them more or less susceptible to interaction with HNF4*α*. It is, therefore, expected that the enhanced PPAR*α*/RXR*α*-mediated transactivation by HNF4*α* only concerns a very limited subset of PPAR*α* target genes that display composite PPREs, similar to those identified in *Thb* and *Mfp-1*.

Lastly, the chromatin structure within the serine protease inhibitor (serpin) gene cluster was found to be orchestrated by the nuclear receptor HNF4*α* and HNF1*α* [56]. Additionally, expression of the chromatin remodeling genes *Smarcd3* and *Cdt-1* was found to be altered in HNF4∆L mice suggesting that these factors are potential candidates that may contribute to the indirect effects of HNF4*α* [57]. In support of this idea, it can be hypothesized that a similar regulation also takes place at the *Thb *gene locus. Supporting this hypothesis, we found that the transactivation of PPAR*α*/RXR*αvia* TB PPRE3 was similarly induced by HNF4*α* or trichostatin A, a well-known inhibitor of histone deacetylation (data not shown).

From a physiological point of view, recent work has shed light on the critical role of HNF4*α* in the control of the expression of enzymes that drive fatty acid *β*-oxidation for energy production in *Drosophila *[58]. Given that PPAR*α* also acts at the level of the *β*-oxidation pathway in mammals, it may come as no big surprise that the PPAR*α* and HNF4*α* signaling routes intersect [31]. To what degree the interaction between the PPAR*α*/RXR*α* heterodimer and HNF4*α* influence peroxisomal lipid catabolism under more physiological conditions deserves further investigation. 

In summary, this study shows that *Thb* is a dual target of the two liver enriched nuclear receptors HNF4*α* and PPAR*α.* Our work also indicates that PPAR*α*/RXR*α* likely contacts HNF4*αvia* RXR*α* and in turn modulates the transcription of *Thb*. Hence, through interaction with other previously bound nuclear receptors to chromatin DNA, HNF4*α* likely facilitates the recruitment of coactivators and may enhance gene transcription.The convergence of the HNF4*α*, PPAR*α*, and RXR*α* signalling pathways underscores the complex interplay involved for the correct transcriptional response of peroxisomal *β*-oxidation.

## Abbreviations

DR1: Direct Repeat-1
HNF4*α*: Hepatocyte nuclear factor-4 alpha
PPAR*α*: Peroxisome proliferator-activated receptor alpha
ThB: 3-ketoacyl-CoA thiolase B
Luc: Luciferase.
